# Safe multidisciplinary approach in deeply infiltrating endometriosis (DIE): is it feasible?

**DOI:** 10.5935/1518-0557.20140020

**Published:** 2014

**Authors:** Ivete de Ávila, Luciana M P Costa, Mario Soto Jr., Ivone D S Filogônio, Márcia M Carneiro

**Affiliations:** 1 Gynecology Clinic Biocor Hospital - Nova Lima/MG - Brazil; 2 Coloprocotology Clinic Biocor Hospital - Nova Lima/MG - Brazil; 3 Urology Clinic Biocor Hospital - Nova Lima/MG - Brazil; 4 Department of Obstetrics and Gynecology, Federal University of Minas Gerais (UFMG) - Belo Horizonte/MG - Brazil

**Keywords:** Embryo transfer, *in vitro* fertilisation, multiple pregnancy, pregnancy, survey

## Abstract

**Objective:**

Evaluate the type and incidence of postoperative complications after surgery for deep infiltrative endometriosis at Biocor Hospital.

**Methods:**

Our observational study involved a multidisciplinary surgical team that performed laparoscopy on 154 patients suffering from pelvic pain. Surgical complications occurring up to the 30^th^ postoperative day were recorded.

**Results:**

Mean age patient age was 34.1 years. Infertility was present in 69 (45%) although 31% had not attempted to get pregnant. Dysmenorrhea was the most frequent symptom (79.3%) followed by chronic pelvic pain (59.7%) and deep dyspareunia (48,7%). Most cases required extensive surgery as the majority (n=117; 76.9%) were classified as severe endometriosis (ASRM grade IV). The most frequent surgical procedures were: 136 adhesiolysis, 100 intestinal surgeries (85 retosigmoidectomies), 92 peritonal lesion excision, 39 vaginal resections, 19 myomectomies, 21 hysterectomies and 5 partial bladder resections. Postoperative complications were recorded in 14 (9.59%) patients: 8 (5.48%) major complications and 6 (4.11%) minor. Major complications included blood transfusion (n=2) retosigmoid anastomosis dehiscence (1), rectovaginal fistula (n=1), urinary fistula (n=1), deep vein thrombosis (n=1), lower limb compartment syndrome with motor deficit (n=1) and one intestinal obstruction (n=1). Minor complications were abdominal wall infection (n=3), peripheral neuropathy (n=3), bladder atony (n=1) and bladder perforation (n=1). No deaths were observed. All major complication cases underwent retosigmoidectomy associated with vaginal resection (n=6), uterosacral ligament excision (n=5) or hysterectomy (n=3).

**Conclusion:**

The surgical treatment of DIE is complex and subject to complications. The surgical expertise of a multidisciplinary team plays a vital role in this setting.

## INTRODUCTION

Deep infiltrative endometriosis (DIE) has been defined as endometriosis that penetrates more than 5 mm under the peritoneal surface (Cornillie *et al.*, 1990). In this situation, endometriotic implants can involve the uterosacral ligaments, the pouch of Douglas (retrocervical endometriosis), the rectovaginal septum, and even the sigmoid colon and rectum. Pain strongly correlates with the depth of the DIE lesions and the radical surgical removal of lesions remains the mainstay of treatment (Koninckx *et al.*, 1991; Porpora *et al.*, 1999; Fauconnier *et al.*, 2002; Chapron *et al.*, 2006). In this setting, the effect of medical treatments in terms of pain relief can be substantial (Vercellini *et al.*, 2009; Mabrouk *et al.*, 2011), but to ensure the complete removal of the disease and obtain the best results in terms of quality of life, extensive surgical removal of endometriotic lesions may be required (Chopin *et al.*, 2005; Seracchioli *et al.*, 2007; Darai *et al.*, 2011; Mabrouk *et al.*, 2011). Several published studies suggest that the complete excision of endometriosis offers good long-term symptomatic relief, especially for those women with severe or debilitating symptoms (Dubernard *et al.*, 2006; Mereu *et al.*, 2007; Stepniewska *et al.*, 2009; Donnez & Squifflet, 2010; Kavallaris *et al.*, 2011; Meuleman *et al.*, 2012). Treatments should be tailored to address the wishes of women according to the specific characteristics of the disease (Leyland *et al.*, 2010; Dunselman *et al.*, 2014). Although many questions remain unanswered, there is evidence to support the use of laparoscopic surgery to improve pain and infertility (Dubernard *et al.*, 2006; Stepniewska *et al.*, 2009; Donnez & Squifflet, 2010). Establishing a systematic strategy is essential to make surgery more reproducible, safer and less time-consuming. Serious complications however may arise and further interfere with quality of life (Ford *et al.*, 2004; Fauconnier & Chapron, 2005; Seracchioli *et al.*, 2007; Donnez & Squifflet, 2010; Fritzer *et al.*, 2012).

Laparoscopic eradication of all visible implants is feasible and safe when surgery is performed in a referral center (Minelli *et al.*, 2010; Fritzer *et al.*, 2012). Unfortunately, even in the most expert hands, complications may occur and further interfere with quality of life and fertility (Donnez & Squifflet, 2010; Ford *et al.*, 2004; Darai *et al.*, 2007; Vercellini *et al.*, 2007; Slack *et al.*, 2007; Minelli *et al.*, 2009; Kovoor *et al.*, 2010; Kondo *et al.*, 2011).

As endometriosis is a chronic disease an integrated approach involving a multi-disciplinary team is not just needed but fundamental. Thus a multidisciplinary surgical team led by a surgically experienced gynecologist working together in complex cases with urologists, gastrointestinal surgeons and/or general surgeons may all play an important role in providing adequate treatment and as well as increasing the likelihood of providing consistent, evidence-based and cost-efficient care (D’Hooghe *et al.*, 2006; Simoens *et al.*, 2012).

As our multidisciplinary surgical team has been working for over a decade (Ávila, 2008), we aimed at evaluating the type and incidence of postoperative complications after surgery for DIE Biocor Hospital.

## MATERIAL AND METHODS

This observational study involved a muldisciplinary surgical team who operated on women sufferering from pelvic pain from Januray 1998 to April 2011. All surgeries were laparoscopies performed by the same gynecologist (IA) and coloproctologist (LMPC). Only cases with histological confirmation of endometriosis were included. Relevant preoperative, intraoperative, and postoperative data were retrieved and recorded in an Excel spreadsheet. Surgical complications occurring up to the 30^th^ postoperative day were recorded.

All women underwent gynaecological examination, pelvic transvaginal and abdominal ultrasonography in order to evaluate the presence of pelvic endometriosis before surgery. Other diagnostic tests were performed when indicated, as previously described (Dunselman *et al.*, 2014). All women were scheduled for laparoscopic management of deep infiltrating endometriosis and they gave informed written consent to surgical treatment and the possible use of their anonymous data for research purposes. The study protocol was approved the local Ethics Committee. The surgical strategy was complete laparoscopic excision of all visually suspected endometriotic lesions and the laparoscopic procedures were performed by the same surgeon (I.A.) and coloproctologist (LMPC). The surgical team had an extensive background in laparoscopic treatment of patients with DIE but we chose to evalute only one surgeon in order to have a uniform criteria for all cases. Laparoscopic resection of endometriosis was performed as previously described (Ávila, 2008; Costa *et al.*, 2010). Segmental recto-sigmoid resection was preformed when bowel function was greatly impaired and when radiological diagnosis of intestinal endometriosis confirmed the presence of intestinal lesions associated with marked restriction of the bowel lumen after colonoscopy. Moreover, deciding the necessity of intestinal resection or intestinal nodule shaving, we took into account endometriosis and intestinal symptoms, impairment of quality of life due to intestinal symptoms, desire of pregnancy and finally the intra-operative evaluation performed by the gynecological surgeon and the coloproctologist. Only after histological confirmation of diagnosis, the patients were included in this analysis. Deep infiltrating endometriosis (DIE) was confirmed when the lesion penetrates >5 mm under the peritoneal surface. We considered intestinal DIE when the lesion infiltrated the muscularis.

## RESULTS

During the study period, laparoscopic resection for DIE was carried out in 154 consecutive women. Table 1 presents patient characteristics. Mean patient age was 34.1 years and the majority (n=117;76.9%) were classified as ASRM (1996) stage IV. Most women (n=69; 45%) were infertile although 31% had not attempted to get pregnant at all. Dysmenorrhea was the most frequent symptom (79.3%) followed by chronic pelvic pain (59.7%) and deep dyspareunia (48.7%). Data analysis showed that 14 (9.59%) patients developed postoperative complications. Laparoscopic procedures are summarized in table 2.

**Table 1:** Patient characteristics

Patient characteristcs (n=154)	N
Mean age	34.1(±5.8) anos
• **Presenting symptoms** • Infertility • Patients who did not attempt pregnancy • Dysmenorrhea • Chronic pelvic pain • Deep dyspareunia • Dyschezia Dysuria	69(45%) 47(31%) 119(79.3%) 89(59.7%) 73(48.7%) 67(44.7%) 18(12%)
ASRM (1996) stage IV	117(76.9%)

**Table 2:** Laparoscopic procedures

Type of procedure	N
• **Gynecological** • Adhesiolysis • Peritoneal endometriotic lesion exeresis • Ovarian cystectomy • Oophorectomy • Vaginal resection • Hysterectomy • Myomectomy	136 92 50 20 39 21 19
• **Intestinal** • Segmental retosigmoidectomy • Retosigmoid disc excision • Appendectomy • Combined resection (disc and segmental retosigmoidectomy)	54 29 15 2
**Urinary** • Partial bladder resection	5

Major complications included blood transfusion (n=2) rectosigmoid anastomosis dehiscence (1), rectovaginal fistula (n=1), urinary fistula (n=1), deep vein thrombosis (n=1), lower limb compartment syndrome with motor deficit (n=1) and one intestinal obstruction (n=1). Minor complications were abdominal wall infection (n=3), peripheral neuropathy (n=3), bladder atony (n=1) and bladder perforation (n=1). No deaths were observed. All major complication cases underwent retosigmoidectomy associated with vaginal resection (n=6), uterosacral ligament excision (n=5) or hysterectomy (n=3).

## DISCUSSION

We report the results of 154 surgeries for DIE performed by our multidisciplinary surgical team which is composed of three gynecological surgeons, one coloproctologist and an urologist as well as reproductive endocrinology and infertility specialist. Surgical management of severe disease often requires a multidisciplinary team approach, because surgery may be complex, requiring input from colorectal and urologic colleagues. In our center we have been practicing complete surgical excision of all affected tissue for more than a decade. Our current multidisciplinary approach to surgery was completely established by the beginning of 2000. Laparoscopic skills of the gynecologists, colorectal surgeons, and urologists involved in the endometriosis team were fully developed by this time. The aim of this study was to examine the short term surgical outcome in this group of patients in terms of the rate of surgical complications.

We decided to include only laparoscopies performed by the same gynecologist (IA) and coloproctologist (LMPC) in order to eliminate possible confounding factors although the other surgeons usually participate actively in all procedures.

The 154 women operated on by our team suffered from pelvic pain from but 45% were also infertile and 31% had not even attempted to get pregnant. They were also young (mean age 34.1 years) so fertility preservation was an important issue for many of them. It is now accepted that effective symptomatic management of DIE requires complete excision of the rectovaginal and intestinal disease (Chopin *et al.*, 2005; Dubernard *et al.*, 2006; Seracchioli *et al.*, 2007; Darai *et al.*, 2011; Stepniewska *et al.*, 2009). Recent published data show that the advancements in laparoscopic surgery have enabled a fertility-sparing approach for this group of patients (Darai *et al.*, 2007; Donnez & Squifflet, 2010; Minelli *et al.*, 2010; Pandis *et al.*, 2010; Douay-Hauser *et al.*, 2011; Wattiez *et al.*, 2013). Thus hysterectomy and removal of both ovaries are not mandatory any more. This fertility-sparing approach is effective in reducing pain and improving quality of life and results in increased fertility rates (Fedele *et al.*, 2004; Dubernard *et al.*, 2006; Leyland *et al.*, 2010; Minelli *et al.*, 2010; Darai *et al.*, 2011).

Most cases required extensive surgery as the majority (n=117; 76.9%) were classified as severe endometriosis (ASRM grade IV). This reflects the establishment of our endometriosis team over the years with increasing numbers of women being referred to us for specialized treatment as well as the reported delay of surgical diagnosis of deep infiltrating endometriosis which is significantly longer for patients with advanced stage IV disease (Matsuzaki *et al.*, 2006). The surgical treatment of deep infiltrative endometriosis is challenging and complex. Currently, the gold standard for patient care is the referral to tertiary centers with a multidisciplinary team including gynecologists, colorectal surgeon and urologist with adequate training in advanced laparoscopic surgery (Simoens *et al.*, 2012). The surgical technique is essential to adequately manage the disease and to minimize the risk of complications. In addition, collaboration between gynecologists, urologists, and colorectal surgeons is of paramount importance for successful management of the case in one surgical intervention providing minor risk of complications, shorter hospital stay, and faster functional recovery (D’Hooghe *et al.*, 2006; Simoens *et al.*, 2012; Dunselman *et al.*, 2014).

Data analysis showed that an overall complication rate of 9.59% and the majority of them could be classified as major complications (n=8; 57.1%). Our rate is compatible with other published studies (Donnez & Squifflet, 2010; Slack *et al.*, 2007; Kondo *et al.*, 2011; De Cicco *et al.*, 2011). Evaluating complications in the surgical treatment of DIE is rather difficult as the majority of the studies are retrospective case series, including only a few women. De Cicco *et al.* (2011) published a systematic review on bowel resection in women suffering from endometriosis with 77% of the studies included contained fewer than 50 participants and only four of the 30 studies presented included more than 100 participants. It is not clear from these smaller studies whether the reported cases reflect the complication rate during the surgeons’learning curve. Since the complication rates vary considerably, it is possible that the results are heavily influenced by the current experience of the surgical team (Wright *et al.*, 2011). We do not believe our results are heavily influenced by our learning-curve as most complications developed over the years and were not clustered in the beginning of our work. Another fundamental point to consider is that our patients were young (mean age 34.1 years) and most (n=69; 45%) were infertile and 31% had not attempted to get pregnant at all. The role of DIE in infertility has not been established so far neither the precise effect of surgery has been defined Somigliana *et al.*, 2009; Pandis *et al.*, 2010; Somigliana *et al.*, 2011). Whereas some consider that conservative surgery for rectovaginal endometriosis in infertile women does not modify the reproductive prognosis although it does increase pain-free survival time while increasing complication rates (Costa *et al.*, 2010; Somigliana *et al.*, 2011). Others, however, state that surgery not only does not affect chances of conception, but results in increased conception rates (Fedele *et al.*, 2004; Donnez & Squifflet, 2010; de Ziegler *et al.* 2010).

The current evidence strongly supports the effectiveness of radical laparoscopic resection in relieving endometriosis-associated symptoms and enhancing psychological well-being. In addition, studies suggest a general improvement of quality of life, however, further studies are needed to support this observation. It must be noted however that extensive surgery for intraperitoneal and deep endometriosis in infertile women does not modify global fertility outcome but is associated with a higher complication rate (Wattiez *et al.*, 2013). A review (Vercellini *et al.*, 2012) of 11 selected studies, the mean post-operative conception rate in all women seeking pregnancy independently of preoperative fertility status and IVF performance was 39% (223/571), but dropped to 24% (123/510) in infertile patients who sought conception spontaneously. The 15% difference is statistically significant. Thus infertile women with rectovaginal endometriosis considering surgery, should be carefully informed of the real probability of post-operative conception avoiding generic overestimations.

We have previously published an evaluation of 98 women with intestinal endometriosis in order to identify the types of surgical procedures performed and the operative morbidity in women with bowel endometriosis. Operative morbidity was observed in 9.2% and major complications were rectovaginal fistula (1%) and anastomosis dehiscence (1%). After a mean follow-up of 14 months that included 42 patients, recurrence of clinical symptoms (pelvic pain and dyspareunia) was observed in 8 cases as well as 4 cases of asymptomatic intestinal wall endometriosis recurrence which was identified by ultrasonography (Costa *et al*., 2010).

Networks and centres of excellence within a multi-disciplinary context is the only way forward to ensure that women with persistent/chronic endometriosis receive consistent, evidence-based and cost-efficient care within a framework which is able to provide excellence, continuity of care, a multidisciplinary approach, research, training and cost effective management (D’Hooghe *et al.*, 2006; Simoens *et al.*, 2012; Dunselman *et al.*, 2014).

Our findings are in agreement with recent published papers which emphasize that a well-trained multidisciplinary team can perform surgical treatment of DIE laparoscopically with low incidence of major complications (Bachmann *et al.*, 2014) though we report on a much larger case series.

## CONCLUSION

DIE affects young women for whom fertility preservation is a major issue. The surgical treatment of DIE is complex and subject to complications. This should be taken into consideration when deciding on treatment. Laparoscopic excision of deeply infiltrating pelvic endometriosis within a multidisciplinary setup in a tertiary referral center appears to be safe with a low rate of significant short-term complications. The surgical expertise of a multidisciplinary team to allow for a fertility-sparing approach plays a vital role in this setting since most women we treated were infertile, had ovarian endometriomas and a third had never attempted pregnancy.
